# Supplementary material for: The structure of mass political belief systems: A network approach to understanding the left-right spectrum
Source: PLoS One. 2025 Oct 22;20(10):e0333595. doi: 10.1371/journal.pone.0333595 (PMC12543193; doi:10.1371/journal.pone.0333595)
Supplement: S1 File — This file contains a detailed methodological rationale, sensitivity analyses, robustness analyses, network replicability, and centrality metrics. (PDF) [file pone.0333595.s001.pdf]

The structure of mass political belief systems:

A network approach to understanding the Left-Right spectrum

Supporting Information

Table of Contents

Section I: Methodological Rationale 4

Section II: Complete Network Comparison Results 12

Table S1. Significant differences in Strength Centrality (S.C.) indices of particular nodes across the three political networks. 13

Table S2. Significant Differences in particular edge-weights across the three political networks. 14

Table S3. Differences in Global Strength and Global Expected Influence (i.e., absolute and raw sum of edge-weights, respectively) across the three political networks. 15

Table S4. Significant differences in Expected Influence (E.I.) indices of particular nodes across the three political networks. 16

Section III: Sensitivity Analyses 17

Figure S1. Replication of main results. We used random sub-samples from each political group (so as to achieve same N = 489). From the figure it can be seen that the network structures, centrality indices, and main NCT results mirrored the ones from the original analysis that used different Ns. 18

Figure S2. Replication of factor structure in the random sub-samples (N = 489). 19

Section IV: Robustness Analyses 20

Stability Analysis 20

Figure S3. Stability of expected influence centrality indices in the political network of left ideology. According to the plot, the CS coefficient is higher than 0.6, suggesting high levels of stability. 21

Figure S4. Stability of expected influence centrality indices in the political network of centre ideology. According to the plot, the CS coefficient is higher than 0.6, suggesting high levels of stability. 22

Figure S5. Stability of expected influence centrality indices in the political network of right ideology. According to the plot, the CS coefficient is higher than 0.6, suggesting high levels of stability. 23

Accuracy Analysis 24

Figure S6. Accuracy analysis in left network. This reveals that the edge-weight estimates (red line) fall within their 95% confidence intervals (grey area) in the left political network, suggesting high accuracy in the detection of edge weight parameters. 25

Figure S7. Accuracy analysis in centre network. This reveals that the edge-weight estimates (red line) fall within their 95% confidence intervals (grey area) in the centre political network, suggesting high accuracy in the detection of edge weight parameters. 26

Figure S8. Accuracy analysis in right network. This reveals that the edge-weight estimates (red line) fall within their 95% confidence intervals (grey area) in the right political network, suggesting high accuracy in the detection of edge weight parameters. 27

Bootstrapped Exploratory Graph Analysis **28**

Figure S9. Factor structure of the left political network (with edges representing factor loadings). 29

Figure S10. Factor structure of the centre political network (with edges representing factor loadings). 30

Figure S11. Factor structure of the right political network (with edges representing factor loadings). 31

Table S5. Results from Confirmatory Factor Analyses (CFAs) on each political network (using WLSM estimator). 32

Section V: Network Replicability **33**

Section VI: Centrality **34**

Figure S12. Betweenness indices: These indices reflect the frequency with which certain nodes (variables) lie in-between two other nodes (variables) in the network. 34

Figure S13. Closeness indices: These indices reflect the extent to which a node (variable) is closely related to the rest of the nodes (variables) in the network. 35

References **36**

Section I: Methodological Rationale

Background Information. The current study sought to investigate the political belief systems of the left, centre, and right political ideology. To do so, network psychometrics were employed (e.g., Epskamp et al., 2018a). The fundamental assumption of network psychometrics is that psychological constructs (in our case: belief systems) represent complex systems that comprise a multitude of components. The relations of these components are viewed as *'mereological,'* meaning that they are constitutive of the overall system——and not merely its common effects (Robinaugh et al., 2014). Thus, psychological phenomena are viewed as emergent properties that arise from the complex interactions of their components.

With regard to belief systems, previous work has attempted to model attitudes (which do contain beliefs along with other components) in this way (e.g., Dalege et al., 2016, 2018). The Causal Attitude Network (CAN) model was the first attempt to systematically model the various elements of attitudes (e.g., feelings, beliefs, and behaviours toward an attitude object) as a network system (see Dalege et al., 2016). The CAN model was conceptualized as an Ising model that modelled attitudinal elements in a binary fashion, with -1 and +1 representing either agreement or disagreement with a particular attitude element, respectively. Dalege et al. (2016) suggested that the typical attitude dynamics (as formulated in the Social Psychology literature since the early 1900s) follow the dynamics of the Ising model, which was initially formulated in statistical mechanics as a way to model atom spins (Brush, 1967). Their theorizing was that the Ising model fulfilled two fundamental assumptions in the attitude literature; first, that there exist direct interactions between attitude elements (which influence one another) and, second, that attitude elements tend to align with one another (i.e., cognitive consistency; Gawronski, 2012). The CAN model has

been recently extended to the Attitudinal Entropy Framework to incorporate some additional axioms——entropy, energy, and temperature (Dalege et al., 2018).

One of these axioms, namely, the Gibb's entropy one, has been of particular interest to cognitive researchers. Gibb's entropy is a measure of the likelihood of the different possible microstates of a given system (Jaynes, 1965). When all states are equally likely, Gibb's entropy is the highest (suggesting higher levels of randomness since all states are equally likely); when only a particular state is possible, Gibb's entropy is the lowest (since the system's behaviour is completely predictable). With regard to attitudinal systems, it has been previously argued that entropy tends to be low, given certain well-established psychological phenomena (see Dalege et al., 2018); for instance, humans' tendency to strive toward cognitive consistency (Gawronski and Strack, 2012), order (Schrödinger, 1944; Kauffman, 1993), and certainty (Hirsh, Mar, and Peterson, 2012). From a theoretical perspective, this makes sense, since it is attitudes with low entropy that would be preferable: predictable and consistent attitudes are overall more useful, since they tend to organize knowledge structures better, promote higher cognitive consistency, and reduce cognitive dissonance (e.g., Dalege et al., 2016).

Previous research exploring some of CAN's/AE's assumptions has revealed interesting dynamics in attitude systems. An early study by Dalege et al. (2017), for instance, showed that the global connectivity of political attitude networks strongly predicted voting decisions in a U.S. cohort. The authors argued that the predictive value of attitude network systems rests on their connectivity level, with more interconnected networks affecting behaviour more strongly. In a related study, Dalege et al. (2019) showed that political interest predicted higher attitudinal network connectivity, suggesting that higher connectivity in attitude systems directly relates to stronger and more durable attitudinal elements. These results echo the

connectivity hypothesis, which is a central tenet of not only the attitude network theory, but also of the network theory of mental disorders (see Borsboom, 2017). This hypothesis suggests that highly interconnected network systems are stronger and more enduring since they reflect low levels of entropy.

In this study we aimed to assess this hypothesis in a cognitive (as opposed to an attitude) system. In particular, using a data-driven approach, we aimed to assess how connectivity varies in the network systems of political beliefs of individuals with different political ideologies. To this end, we assigned participants into one of three groups: left, centre, and right ideology. We then estimated Gaussian Graphical Models—as opposed to Ising network models—for each of these groups and assessed how certain microscopic and macroscopic network properties varied across these groups. Although global network connectivity was the main measure of interest, we also assessed other network properties including, for instance, the networks' factor (or else community) structure and microscopic connectivity differences across networks. What follows is an outline of our methodology to explicate our reasoning for each of these assessments.

Methodological Details. First and foremost, it is worth noting that, unlike previous studies, this study investigated network dynamics from a Gaussian model, not an Ising model, perspective. The reason for our choice of Gaussian Graphical Model is threefold. Firstly, the current belief variables were measured on an ordinal scale; recoding them into a binary fashion would result in a substantial loss of information (and variation), especially since they were measured on a five-point scale (and the middle ground of belief endorsement would be entirely lost).

Secondly, recent research has suggested that the input in Ising network models matters when it comes to its underlying dynamics (see Haslbeck et al., 2021). In particular, attitude (or related belief) elements that could be endorsed negatively or positively need to be coded into -1 or +1, respectively; whereas other variables (such as psychopathology symptoms) that can be either present or absent must be coded in a 1 or 0 fashion (Finnemann et al., 2021). Currently, the *eLasso* procedure of various R packages (such as *mgm*, *IsingFit* or *rIsing*) does not allow (explicitly) for the former coding. [And although conversion methods between the two coding ways exist, e.g., *IsingFit:: LinTrasform ()*, they are not yet (fully) validated (Kruis, 2020).]

Finally, from a theoretical point of view, it could be argued that individual beliefs are better captured in a Likert-like manner. Indeed, recent commentaries of the Ising model have noted that its attractiveness lies primarily in its simplicity (e.g., Finnemann et al., 2021). From this perspective, more complex models that require more response categories in their variables may be more preferable from a complexity (as well as comprehensiveness) point-of-view.

It is also worth pointing out that, with regard to network connectivity, we have chosen to (primarily) assess the absolute levels of network connectivity. Given the coding of our items, negative correlations were expected between certain political beliefs across networks. Further, regardless of the coding strategy, a positive manifold (i.e., only positive associations within the networks) was not achievable——because even if one were to code all political beliefs such that they were all positive in the left network, negative associations would still be expected in the right network (and vice versa). Nonetheless, based on theory, we would expect both positive and negative associations to be stronger in the political networks of

those on the left and those on the right, compared to those on the centre (see description of Converse's account on mass beliefs in the paper's introduction). For this reason, we have sought to evaluate the connectivity of networks in absolute terms first, and in non-absolute (or raw) terms secondarily (please see next Supplementary section for full details on Network Comparisons).

A final methodological but also conceptual point worth outlining now concerns the way by which we have categorized individuals into left, centre, and right. In particular, a single item asking our respondents to rate themselves on a 10-point Likert scale where they stand regarding the left-right spectrum was used and was trichotomized accordingly to separate individuals into those who stand on the (extreme) left and right and those who stand in the middle (4, 5, 6, 7). In the main paper, we note the limitations of this approach and highlight ways by which such classification strategies can be improved in future research.

Permutation Testing. This study made use of the permutation testing procedure embedded in the Network Comparison Test (NCT) for the statistical examination of differences between the three networks. This procedure entails three steps. First, the observed datasets are analysed to estimate a particular network model and derive the test statistics of interest. In our case, we had three comparison groups and we estimated one Gaussian Graphical Model (GGM) for each. Our GGMs were fitted using the widely-employed and well-validated EBIC glasso procedure (Extended Bayesian Information Criterion) (graphical least absolute shrinkage and selector operator) (please see Epskamp and Fried, 2018). **[**This approach employs *l*1 regularization (i.e., lasso; see Tibshirani, 2011) to penalize the parameter estimates (which in this case are partial correlation coefficients, derived from the precision matrix):

$$\hat{\theta} = arg\ min_{\theta}\{-L(\theta, X) + \lambda \sum_{j=1}^{J} |\theta_j|\}$$

where *J* is the length of our parameter vector ϑ and λ is the regularized (tuning) parameter that determines the level of penalization of our parameter vector (i.e., precision matrix).

Using LASSO, we can ensure that only the most robust parameters remain in the final statistical models. This is because LASSO penalizes small parameter estimates, thereby leading some to zero and reducing the probability of conducting Type I errors (i.e., false-positives). To select the optimal value of the tuning parameter lambda (λ), we chose to reduce the Extended Bayesian Information Criterion (EBIC; Chen and Chen, 2008):

$$EBIC_{\gamma}(\hat{\theta}) = -2L(\hat{\theta}) + \hat{z}_0 \log(N) + 4\gamma \hat{z}_0 \log(P)$$

where *L* is the log-likelihood; $\hat{z}_0$ is the number of non-zero edges; and γ (gamma) is the hyper-parameter (the value of which needs to be manually selected). In our case, we set the gamma value to 0.5 (a conservative value) in order to 'err' more on the side of caution; that is, in order to exclude more parameters and yield a sparser network.

By attenuating our edge-weights (i.e., partial correlations among beliefs) and excluding very small ones (using the abovementioned procedure), we achieve two aims. First, we screen for a sparser network structure, which is more easily interpretable. Second, we ensure that all present edges are likely to be true positives; that is, we achieve high *specificity*, at the cost of *sensitivity*. This trade off is preferable in our case, given the novelty of our analyses.**]**

Second, the reference distribution is created by (a) randomly resampling the data from each comparison group (without replacement); (b) estimating the GGMs again; and (c)

deriving the statistics of interest from them. This procedure was repeated 1,000 times, resulting in a final null-hypothesis sampling distribution (that is, a distribution of random networks that represent the null hypothesis of ‘no actual differences’ across the networks).

Finally, the original test statistic (from the observed data) is pinned against the reference distribution (from the null, sampled data) to assess its extremeness. The resulting *p* value from this comparison has a clear interpretation: It is *‘the proportion of resampled test statistics that are at least as extreme as the observed statistic’* (see van Borkulo et al., 2022). The alpha level was chosen to be 0.01 in order to adjust for multiple (in particular, three) comparisons.

Section II: Complete Network Comparison Results

To avoid cluttering, the online article reported only the Bonferroni-adjusted *p* values for the main network comparison analyses. In this supplementary section, we report the complete set of results, which depict the unadjusted, FDR adjusted, as well as Bonferroni adjusted p values. These p values are, first and foremost, reported for the main results regarding strength centrality comparisons (Table S1); as well as, second, for three additional supplementary analyses. These include: (1) edge-weight comparison tests (i.e., permutation tests, examining whether particular edge-weights differed across the three networks) (Table S2); (2) global expected influence tests (i.e., counterpart of global strength, taking into consideration the raw values of edge-weights) (Table S3); and (3) local expected influence (or centrality) tests (i.e., the counterpart of strength centrality) (Table S4). These tables outline significant results (with various adjustment forms). For the complete set of results (including non-significant ones, at an alpha > .05), the reader is referred to the OSF repository of this research project: https://osf.io/uxa32/.

Table S1. Significant differences in Strength Centrality (S.C.) indices of particular nodes across the three political networks.

	P Value		
Variable	Centre – Left	Centre – Right	Left – Right
P1 (Death Penalty)	.019	-	.025
P2 (Spending Money on the Army)	-	.002*	.003*
P4 (Stiff Jail Terms for Criminals)	-	-	.016
P5 (Euthanasia)	<.001**	-	-
P6 (Gay Rights)	.032	.002*	<.001**
P7 (Higher Benefits for the Poor)	-	.011	.022
P10 (Lower Taxes)	-	-	.002*
P11 (International Government)	.021	-	<.001**
P16 (Brexit)	.003*	.005*	-
P17 (Public Demonstrations)	-	<.001*	<.001*
P18 (International Aid)	-	-	.02

Note: *Remained significant after FDR correction. **Remained significant after Bonferroni correction.

Table S2. Significant Differences in particular edge-weights across the three political networks.

Edge Weight		*P* Value		
Variable 1	Variable 2	Centre – Left	Centre – Right	Left – Right
P2 (Spending money on Army)	P3 (Multiculturalism)	.016	-	-
P1 (Death Penalty)	P5 (Euthanasia)	<.001**	-	.007
P6 (Gay Rights)	P7 (Higher Benefits for Poor)	.028	-	<.01*
P7 (Higher Benefits for Poor)	P8 (Immigration)	.03	-	-
P2 (Spending money on Army)	P9 (Legalized Abortion)	.028	-	.007
P8 (Immigration)	P9 (Legalized Abortion)	.04	-	-
P7 (Higher Benefits for Poor)	P10 (Lower Taxes)	<.001**	-	-
P9 (Legalized Abortion)	P10 (Lower Taxes)	.004	-	.047
P1 (Death Penalty)	P11 (International Government)	.036	-	-
P2 (Spending money on Army)	P12 (Rehabilitation of Offenders)	.046	-	.007
P5 (Euthanasia)	P13 (Traditional Family Values)	.024	-	-
P7 (Higher Benefits for Poor)	P13 (Traditional Family Values)	.001*	-	<.01*
P12 (Rehabilitation of Offenders)	P13 (Traditional Family Values)	.012	-	.009
P7 (Higher Benefits for Poor)	P14 (Monogamy)	.029	-	-
P3 (Multiculturalism)	P16 (Brexit)	.02	-	-
P11 (International Government)	P17 (Public Demonstrations)	.029	-	-
P6 (Gay Rights)	P15 (Trans Rights)	<.001**	<.001**	-
P16 (Brexit)	P18 (International Aid)	-	.028	-
P1 (Death Penalty)	P3 (Multiculturalism)	-	<.01*	-
P1 (Death Penalty)	P8 (Immigration)	-	.024	-
P6 (Gay Rights)	P8 (Immigration)	-	.014	-
P1 (Death Penalty)	P9 (Legalized Abortion)	-	.045	-
P7 (Higher Benefits for Poor)	P10 (Lower Taxes)	-	.022	-
P1 (Death Penalty)	P7 (Higher Benefits for Poor)	-	.02	.01
P2 (Spending money on Army)	P7 (Higher Benefits for Poor)	-	.028	.028
P4 (Stiff Jail Terms)	P7 (Higher Benefits for Poor)	-	<.01*	.004
P10 (Lower Taxes)	P12 (Rehabilitation of Offenders)	-	.016	<.001**
P2 (Spending money on Army)	P14 (Monogamy)	-	<.01*	.025
P4 (Stiff Jail Terms)	P16 (Brexit)	-	.006	<.01*
P7 (Higher Benefits for Poor)	P16 (Brexit)	-	.001	<.001**
P1 (Death Penalty)	P17 (Public Demonstrations)	-	.002	<.001**
P10 (Lower Taxes)	P18 (International Aid)	-	<.001**	<.001**
P11 (International Government)	P17 (Public Demonstrations)	-	-	<.001**
P3 (Multiculturalism)	P4 (Stiff Jail Terms)	-	-	.03
P2 (Spending money on Army)	P6 (Gay Rights)	-	-	.02
P5 (Euthanasia)	P6 (Gay Rights)	-	-	.03
P5 (Euthanasia)	P7 (Higher Benefits for Poor)	-	-	.02
P3 (Multiculturalism)	P9 (Legalized Abortion)	-	-	.04
P4 (Stiff Jail Terms)	P9 (Legalized Abortion)	-	-	.01
P7 (Higher Benefits for Poor)	P9 (Legalized Abortion)	-	-	.004
P6 (Gay Rights)	P17 (Public Demonstrations)	-	-	.02

Note: *Remained significant after FDR correction. **Remained significant after Bonferroni correction.

Table S3. Differences in Global Strength and Global Expected Influence (i.e., absolute and raw sum of edge-weights, respectively) across the three political networks.

$\Delta GS_{LC} = .72$, $pGS_{LC} = .02$<br>$\Delta GEI_{LC} = .74$, $pGEI_{LC} < .01$
$\Delta GS_{CR} = 1.59$, $pGS_{CR} < .01$<br>$\Delta GEI_{CR} = 1.45$, $pGEI_{CR} < .01$
$\Delta GS_{LR} = 0.72$, $pGS_{LR} = .24$<br>$\Delta GEI_{LR} = 0.09$, $pGEI_{LR} = 0.98$

Table S4. Significant differences in Expected Influence (E.I.) indices of particular nodes across the three political networks.

	P Value		
Variable	Centre – Left	Centre – Right	Left – Right
P1 (Death Penalty)	-	<.001**	<.001**
P2 (Spending money on Army)	-	.049	.03
P5 (Euthanasia)	<.001**	-	<.001**
P6 (Gay Rights)	.008	<.001**	-
P10 (Lower Taxes)	-	-	.003*
P11 (International Government)	-	-	<.001**
P12 (Rehabilitation of Offenders)	-	-	<.01*
P16 (Brexit)	-	.001**	<.001**
P17 (Public Demonstrations)	-	.026	.012
P18 (International Aid)	-	.043	-

Note: *Remained significant after FDR correction. **Remained significant after Bonferroni correction.

Section III: Sensitivity Analyses

Since the Network Comparison Test (NCT) is sensitive to sample size imbalances between the comparison groups, a sensitivity analysis was conducted to assess whether the initial patterns (identified with the original comparison groups) can be replicated using randomly sampled and equally sized comparison groups. To do so, random samples were extracted so as to match the smallest group's size (N = 489); Gaussian Graphical Models (GGMs) using the original method of estimation were computed; and all network comparisons were repeated. The results of these procedures are visualized in Figures S1 and S2. The results are further outlined in the repository online at OSF (https://osf.io/uxa32/). In sum, the patterns of this sensitivity analysis converged with those of the original analysis, suggesting no bias from potential sampling variability.

A similar sensitivity analysis was conducted to explore whether our results could be obtained if the centrist group were to be defined in terms of response categories 5-6 (as opposed to 4-7, which we explored in our manuscript). Conducting this network comparison, we replicated the same main result of our work: namely, that both the left-wing (GS_L=25.96; ΔGS_{LC} = 6.34, p < .001) and the right-wing (GS_R=25.86; ΔGS_{RC} = 6.23, p < .001) networks exhibited greater absolute levels of connectivity than the centrist network (GS_C=19.62). Notably, the left-wing and right-wing networks were strikingly similar in their absolute levels of network connectivity (ΔGS_{LR}=0.10, p=.94). The same results held for raw network connectivity: GS_L=20.75, ΔGS_{LC} = 2.75, p = .004; GS_R=21.80, ΔGS_{RC} = 3.80, p < .001; GS_C=18.00; and ΔGS_{LR}=1.05, p=.17. These results imply that our findings are robust across various sub-samples and definitions of the centrist, left-wing, and right-wing groups.

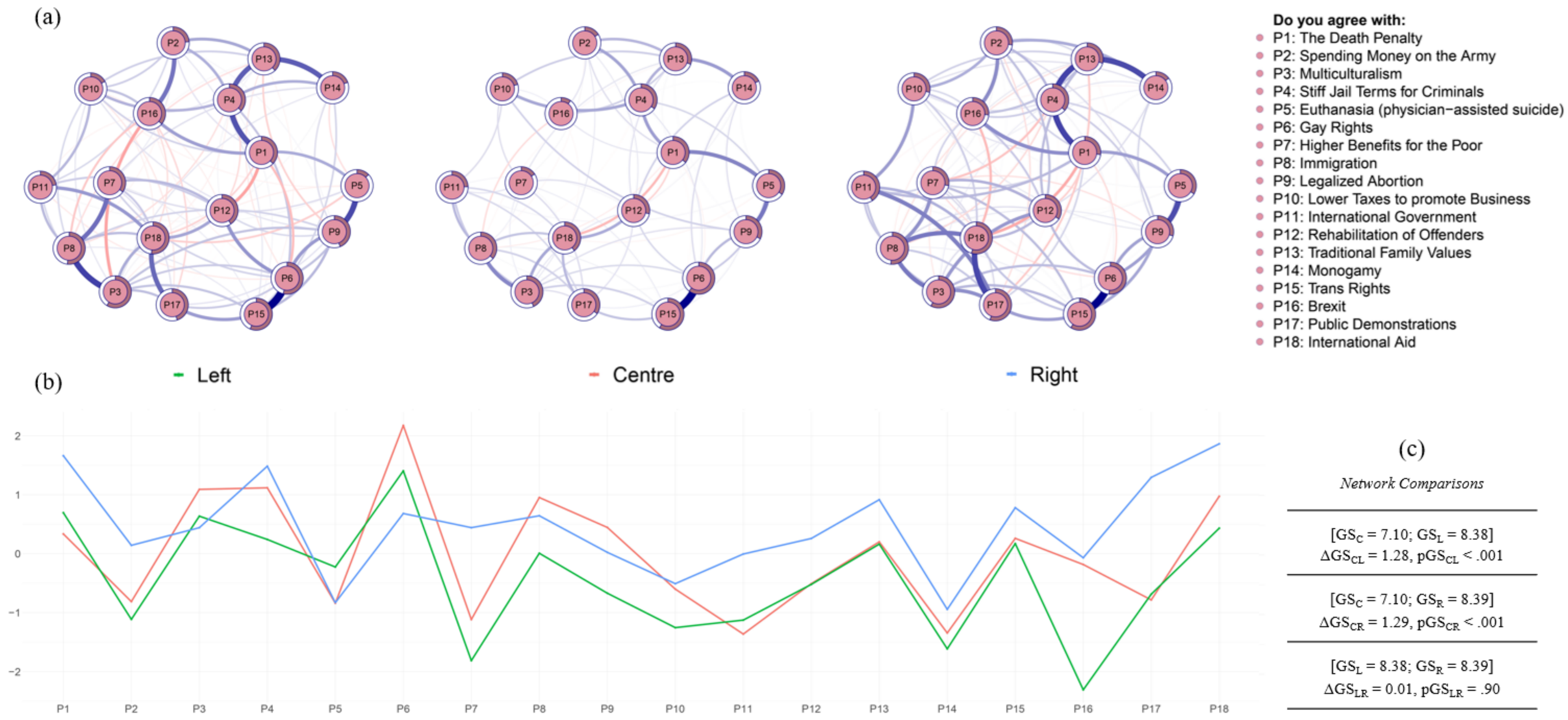

Figure S1. Replication of main results. We used random sub-samples from each political group (so as to achieve same N = 489). From the figure it can be seen that the network structures, centrality indices, and main NCT results mirrored the ones from the original analysis that used different Ns.

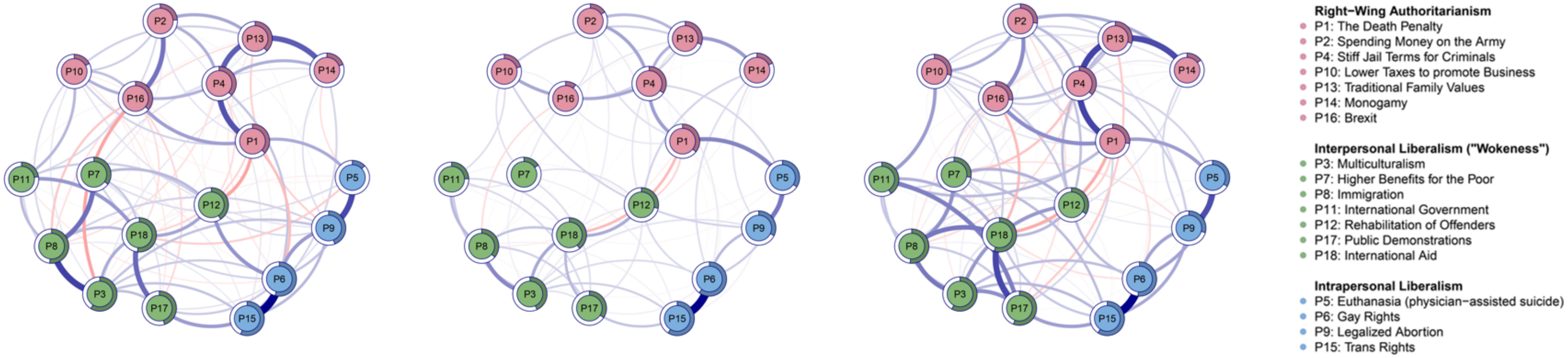

Figure S2. Replication of factor structure in the random sub-samples (N = 489).

Section IV: Robustness Analyses

Stability Analysis

Following the methodological procedures outlined by Epskamp, Borsboom, and Fried (2018b), the R package '*bootnet*' was employed to examine the stability of the Strength and Expected Influence centrality indices. In particular, using the *case-dropping bootstrap* procedure, the main network models were re-estimated with fewer cases and a correlation stability coefficient (*CS-coefficient*) was calculated to quantify the stability of the indices over 1,000 bootstrapped samples. The CS-coefficient reflects the maximum proportion of cases that can be dropped so that the original centrality indices can keep a correlation of at least 0.7 with the bootstrapped centrality indices. The CS-coefficient must be at least 0.25 and ideally larger than 0.5 (Epskamp et al., 2018). This statistical analysis was performed using 1,000 iterations.

The figures below (namely, figures S3, S4, S5) outline the stability of our centrality indices. In sum, our figures reveal that for all political networks, the CS-coefficient was above 0.5; in particular (from left to right): $CS_L = 0.67$; $CS_C = 0.75$; $CS_R = 0.67$.

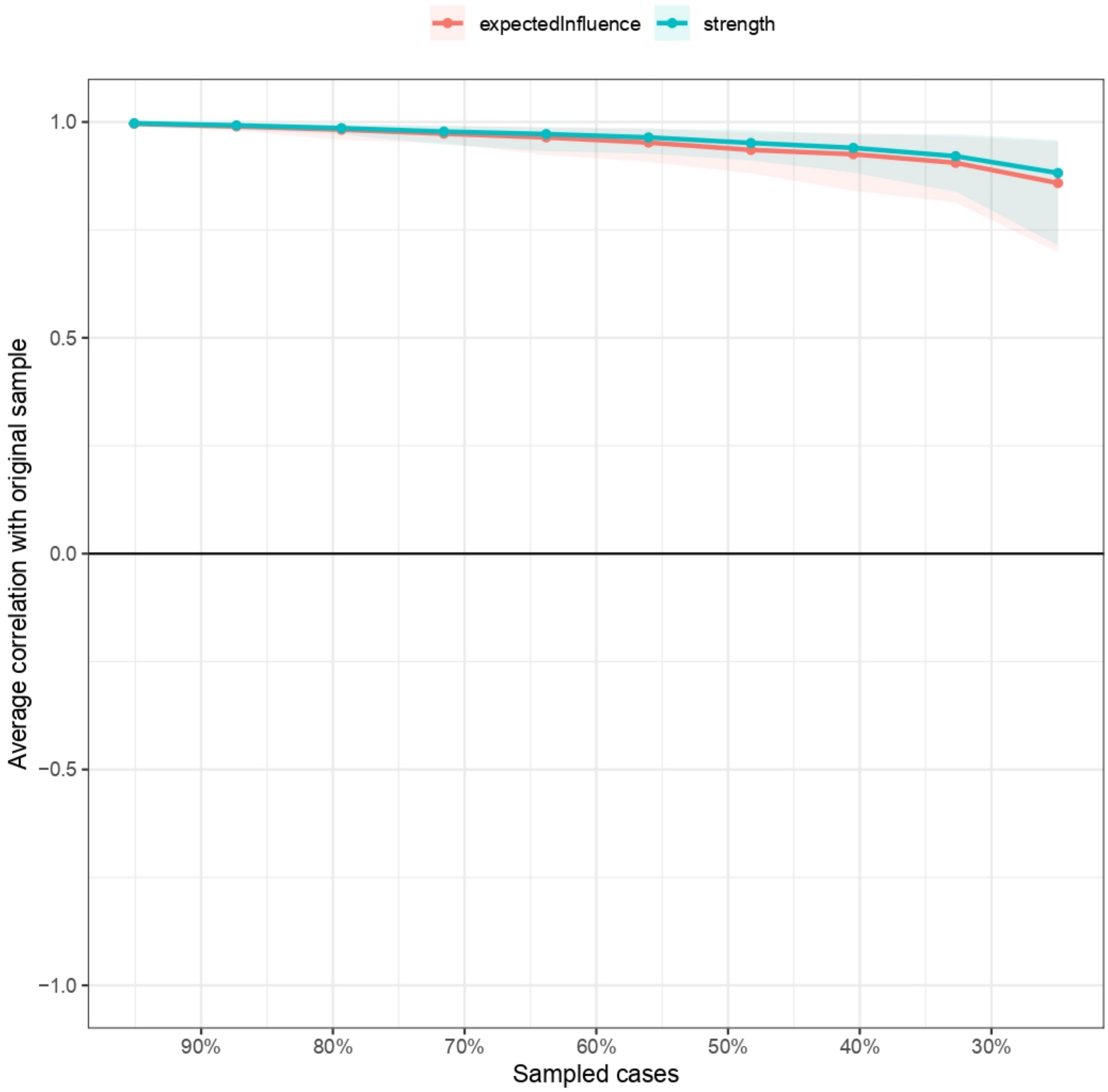

Figure S3. Stability of expected influence centrality indices in the political network of left ideology. According to the plot, the CS coefficient is higher than 0.6, suggesting high levels of stability.

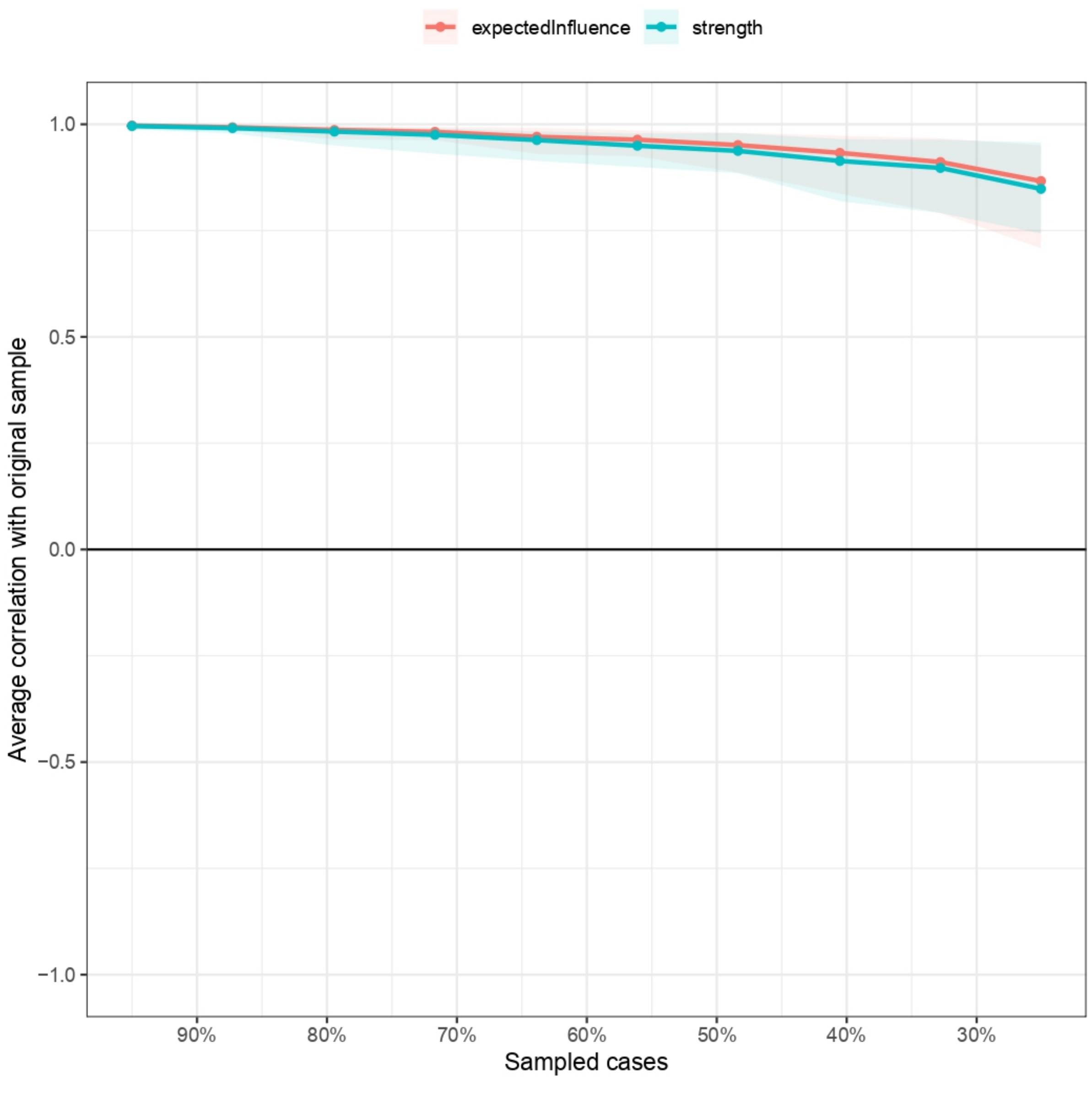

Figure S4. Stability of expected influence centrality indices in the political network of centre ideology. According to the plot, the CS coefficient is higher than 0.6, suggesting high levels of stability.

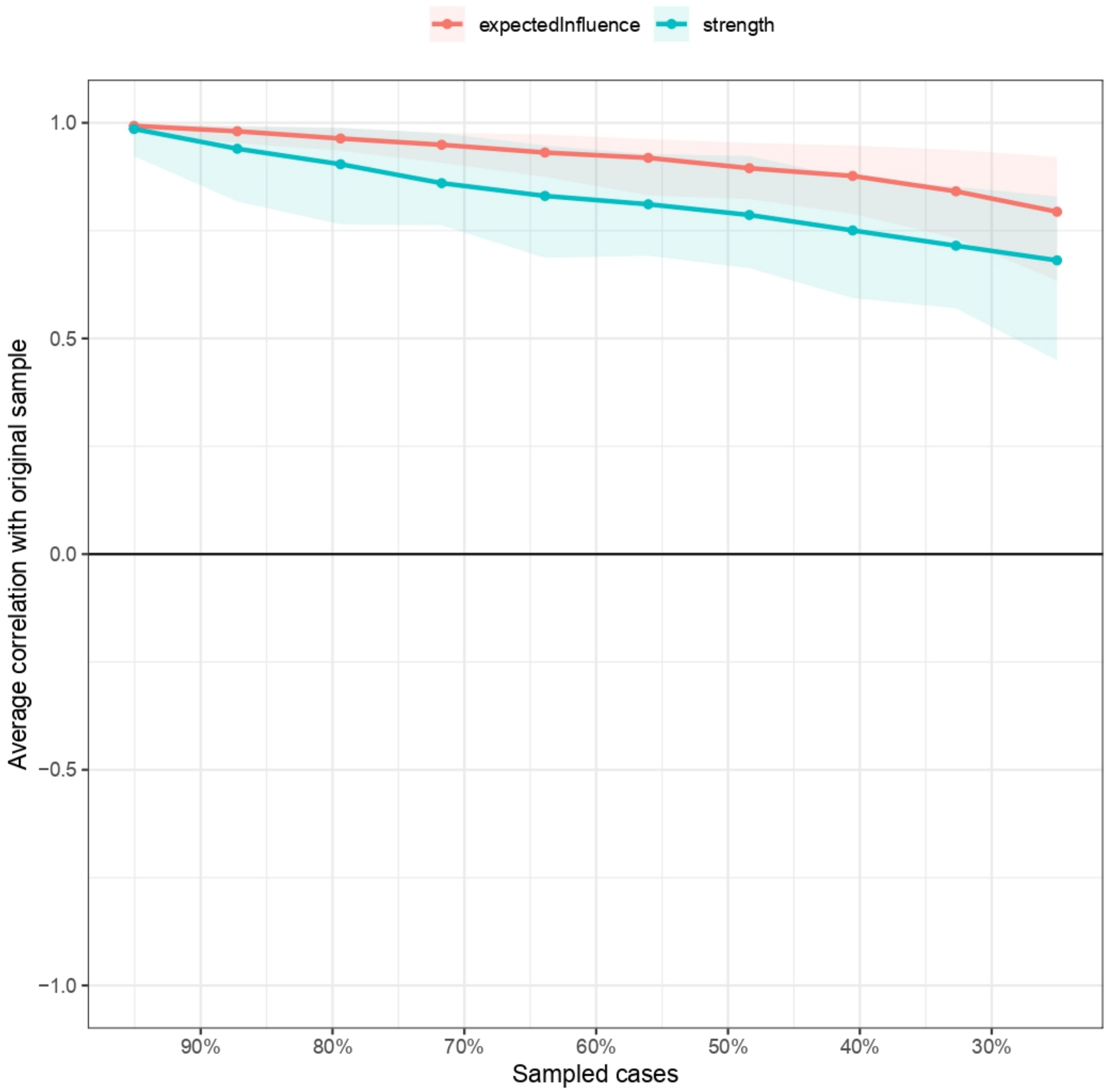

Figure S5. Stability of expected influence centrality indices in the political network of right ideology. According to the plot, the CS coefficient is higher than 0.6, suggesting high levels of stability.

Accuracy Analysis

The R package '*bootnet*' was further used to examine the accuracy of the edge-weights of the three political networks (Epskamp et al., 2018b). In particular, a non-parametric bootstrapped procedure was employed by which the original networks are continually re-estimated with the use of resampling. This procedure ran on 1,000 iterations in order to create confidence intervals through which to judge the accuracy of our sample parameters.

The figures below (namely, figures S6, S7, and S8) represent the resulting confidence intervals from this procedure. The grey area indicates the bootstrapped C.I.s; the red line shows the values of the actual samples. The horizontal lines on the y-axis represent all the edges of the network; their names have been excluded to avoid cluttering. Our analyses here revealed small and accurate confidence intervals for all edge weights (red lines lie within the grey area), suggesting that they represent the population statistics closely.

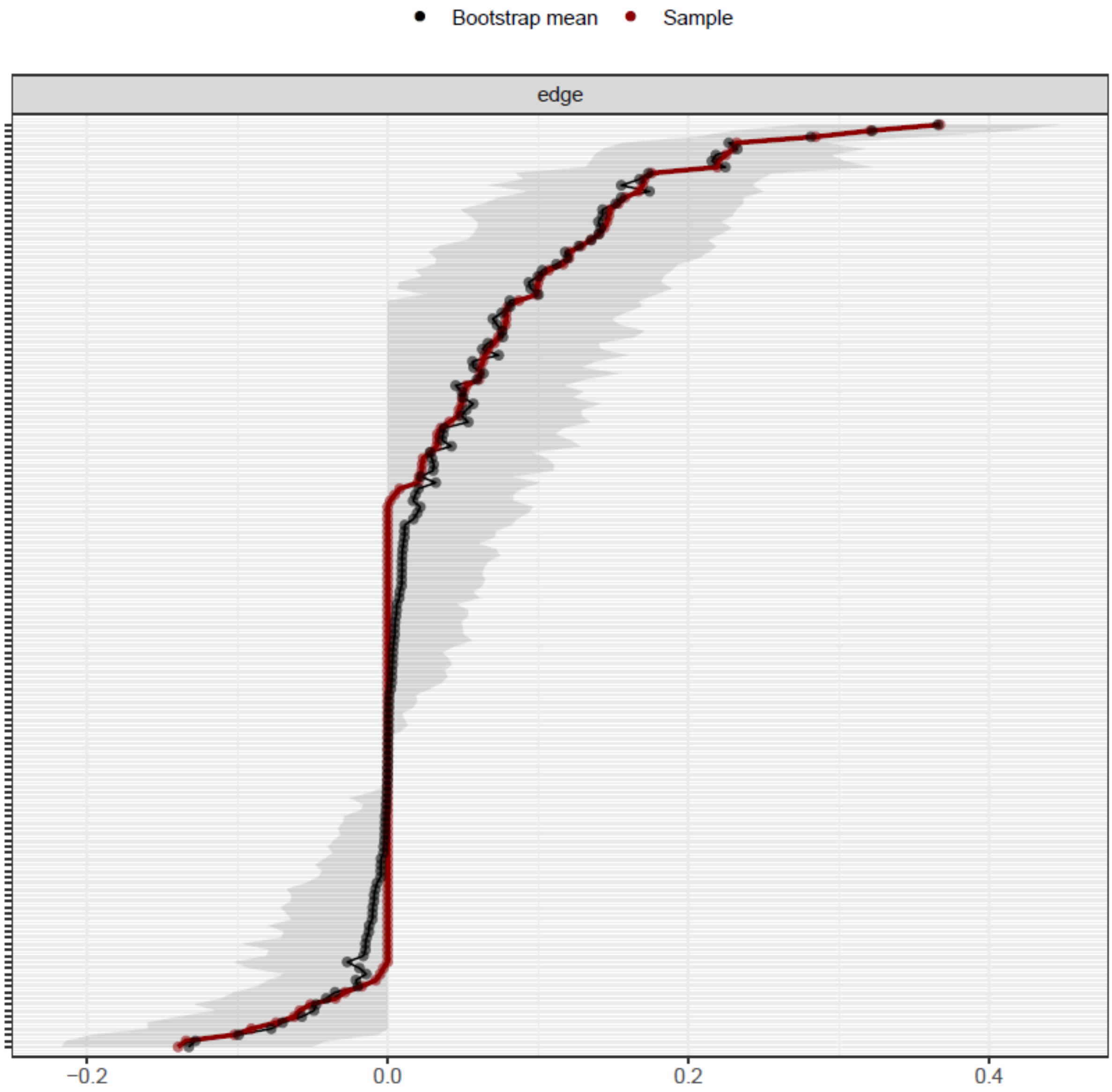

Figure S6. **Accuracy analysis in left network.** This reveals that the edge-weight estimates (red line) fall within their 95% confidence intervals (grey area) in the left political network, suggesting high accuracy in the detection of edge weight parameters.

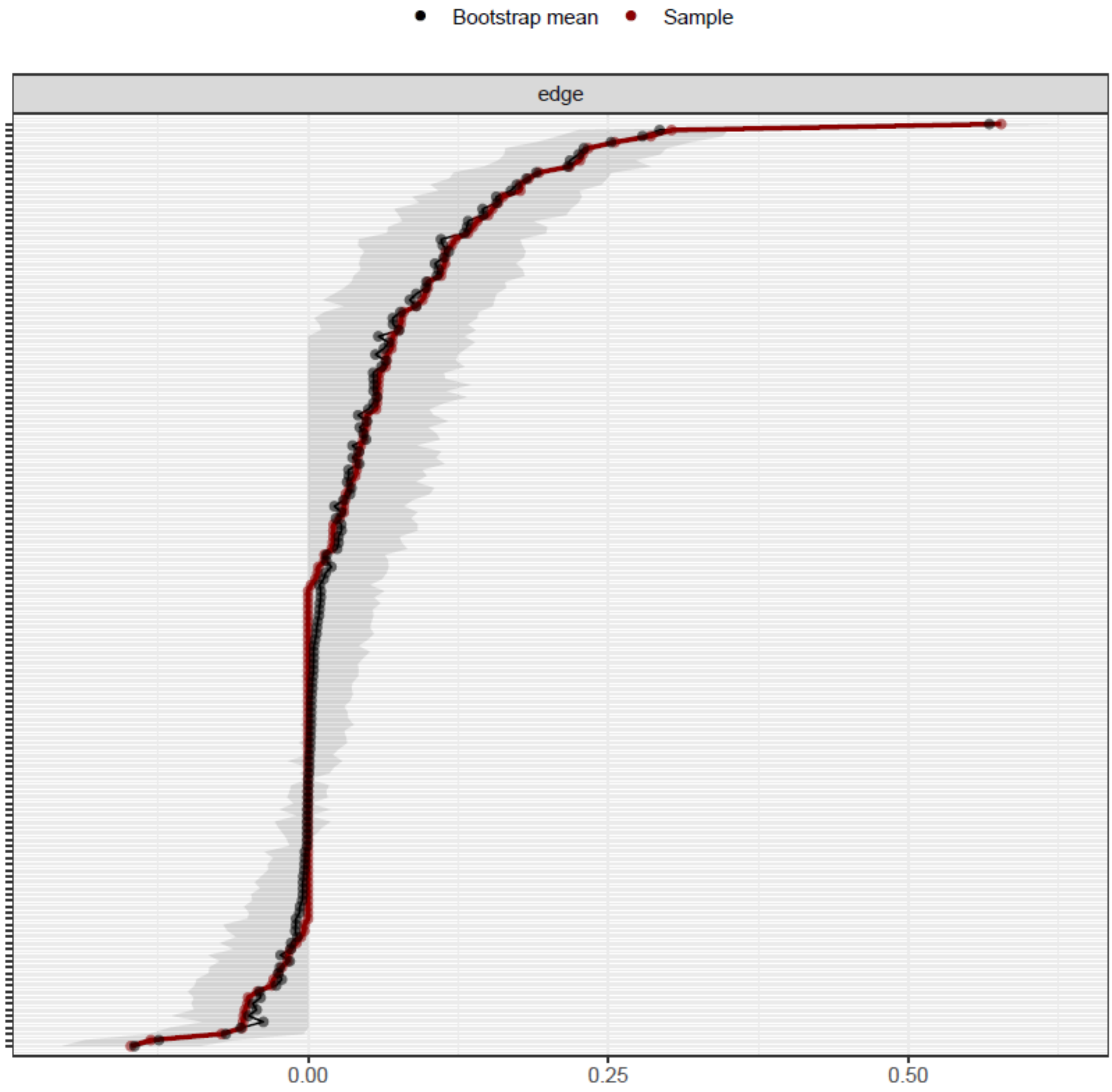

Figure S7. Accuracy analysis in centre network. This reveals that the edge-weight estimates (red line) fall within their 95% confidence intervals (grey area) in the centre political network, suggesting high accuracy in the detection of edge weight parameters.

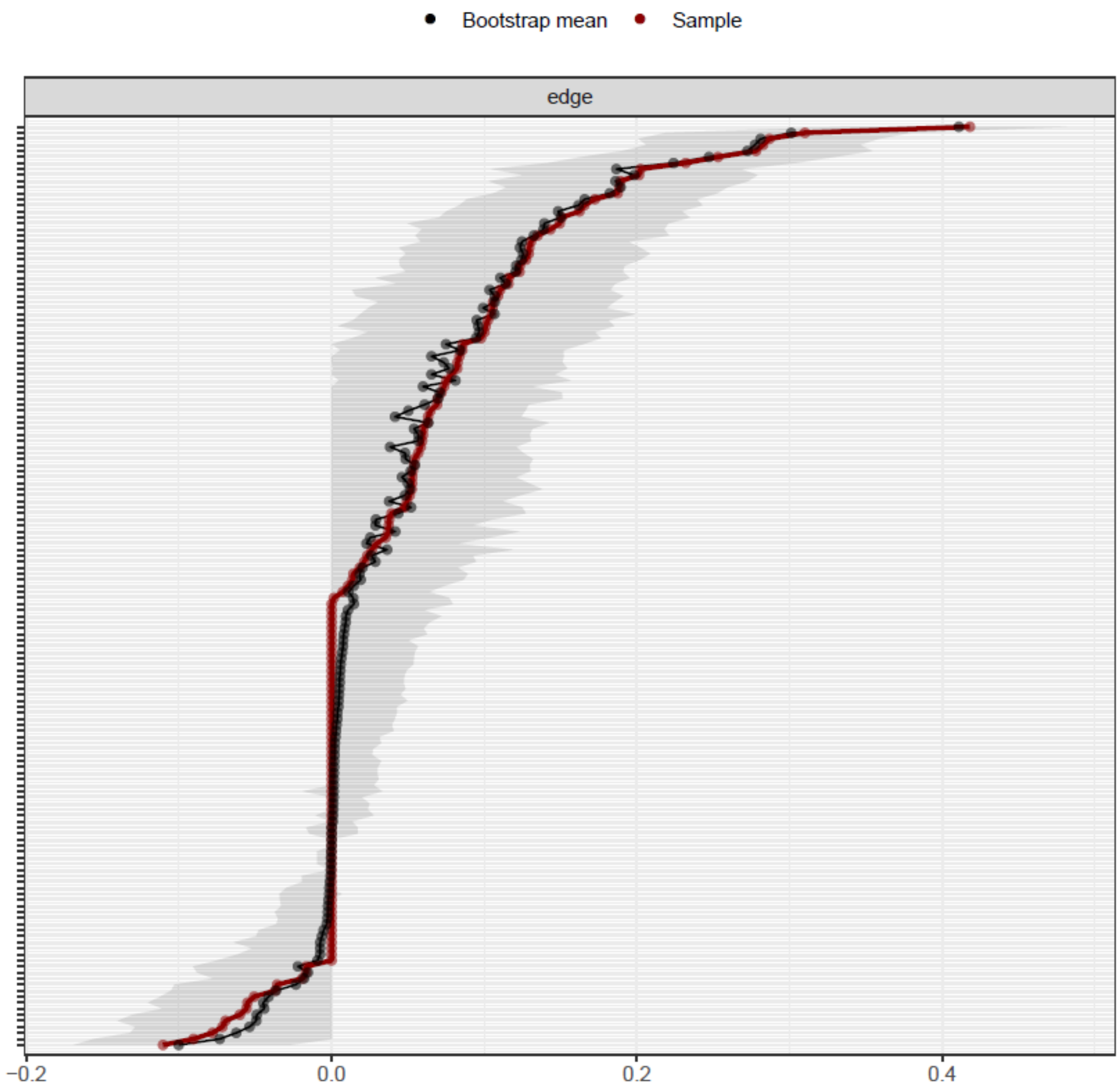

Figure S8. Accuracy analysis in right network. This reveals that the edge-weight estimates (red line) fall within their 95% confidence intervals (grey area) in the right political network, suggesting high accuracy in the detection of edge weight parameters.

Bootstrapped Exploratory Graph Analysis

A new bootstrapping procedure has emerged to assess the stability of the typical Exploratory Graph approach to identifying communities in networks. This approach, namely, bootstrapped Exploratory Graph Analysis (boot EGA), re-estimates the network structure (using the typical EBIC glasso approach; see Section I) using either a parametric or a non-parametric procedure, and conducts an Exploratory Graph Analysis (EGA) on the resulting network to reveal its factor structure (Christensen and Golino, 2019). The procedure is repeated a number of times with the outcome being a distribution of replica networks. From this distribution, a number of note-worthy statistics can be derived, such as the median (or typical) network structure within the distribution of replica networks, the frequency of occurrence of a particular factor structure in the distribution of replica networks, and confidence intervals, to name a few.

In this case, a boot EGA with non-parametric (resampling) procedure was employed, with the number of iterations being set to 1,000. The results validated the original community structure but also revealed that one community (namely, liberalism) could be split into two sub-communities: intra- and inter-personal liberalism. This factor solution (of three, as opposed to two communities) was deemed as more appropriate since it was the most frequently occurring solution in the distribution of replica networks (72.5% – 86% prevalence), suggesting higher levels of stability (due to increased replicability). Furthermore, the typical (or median) network structure also replicated this three-factor solution.

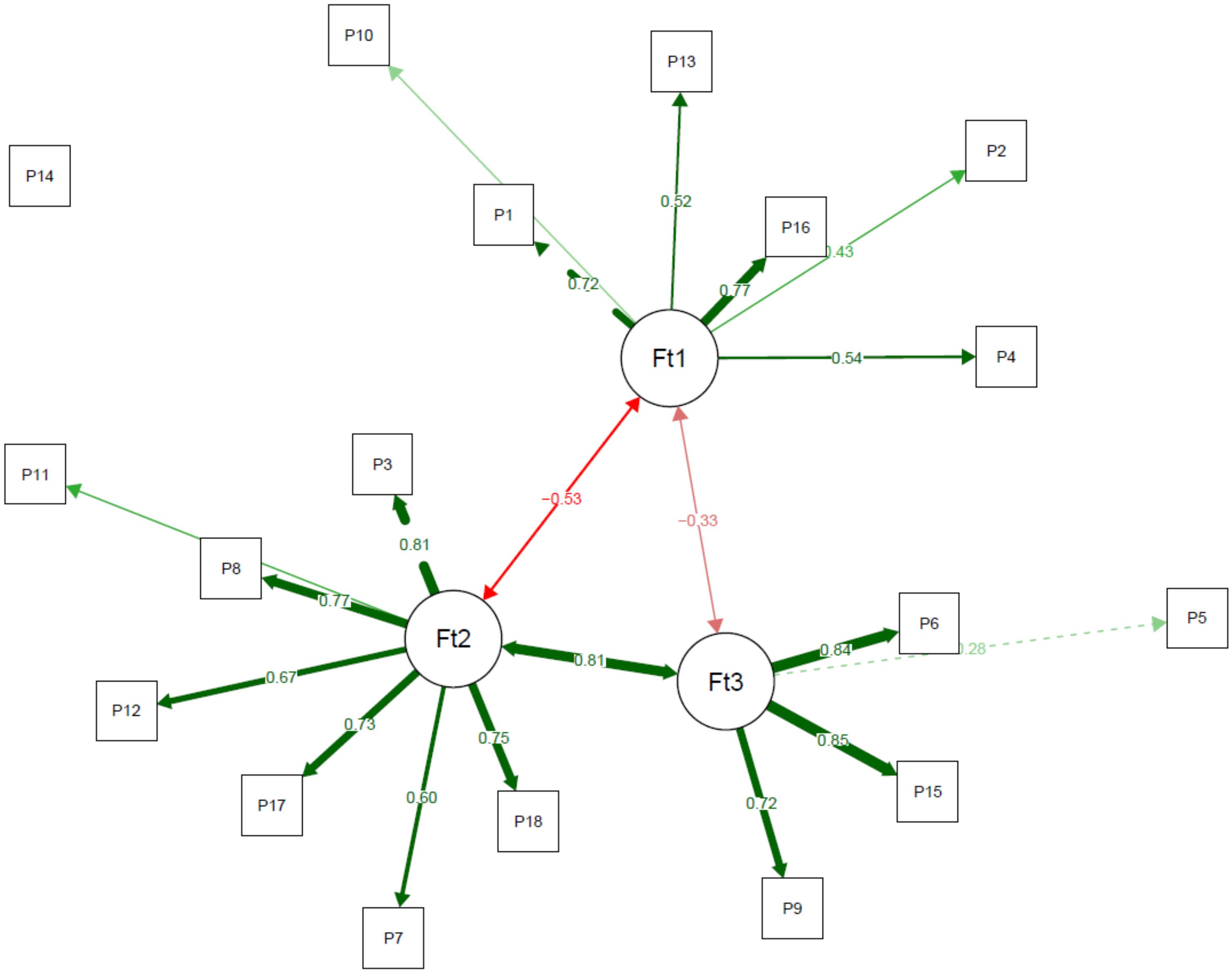

Figure S9. Factor structure of the left political network (with edges representing factor loadings).

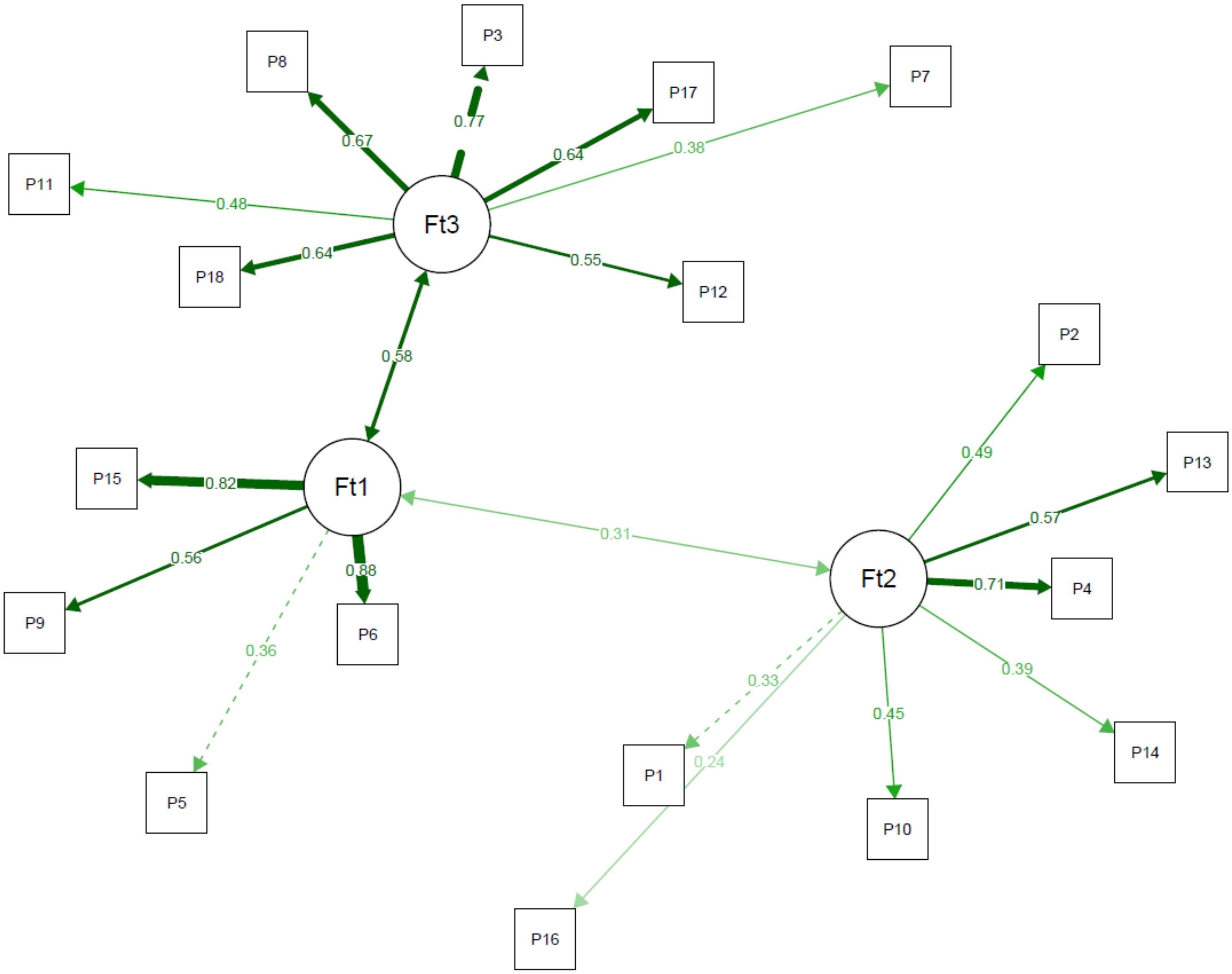

Figure S10. Factor structure of the centre political network (with edges representing factor loadings).

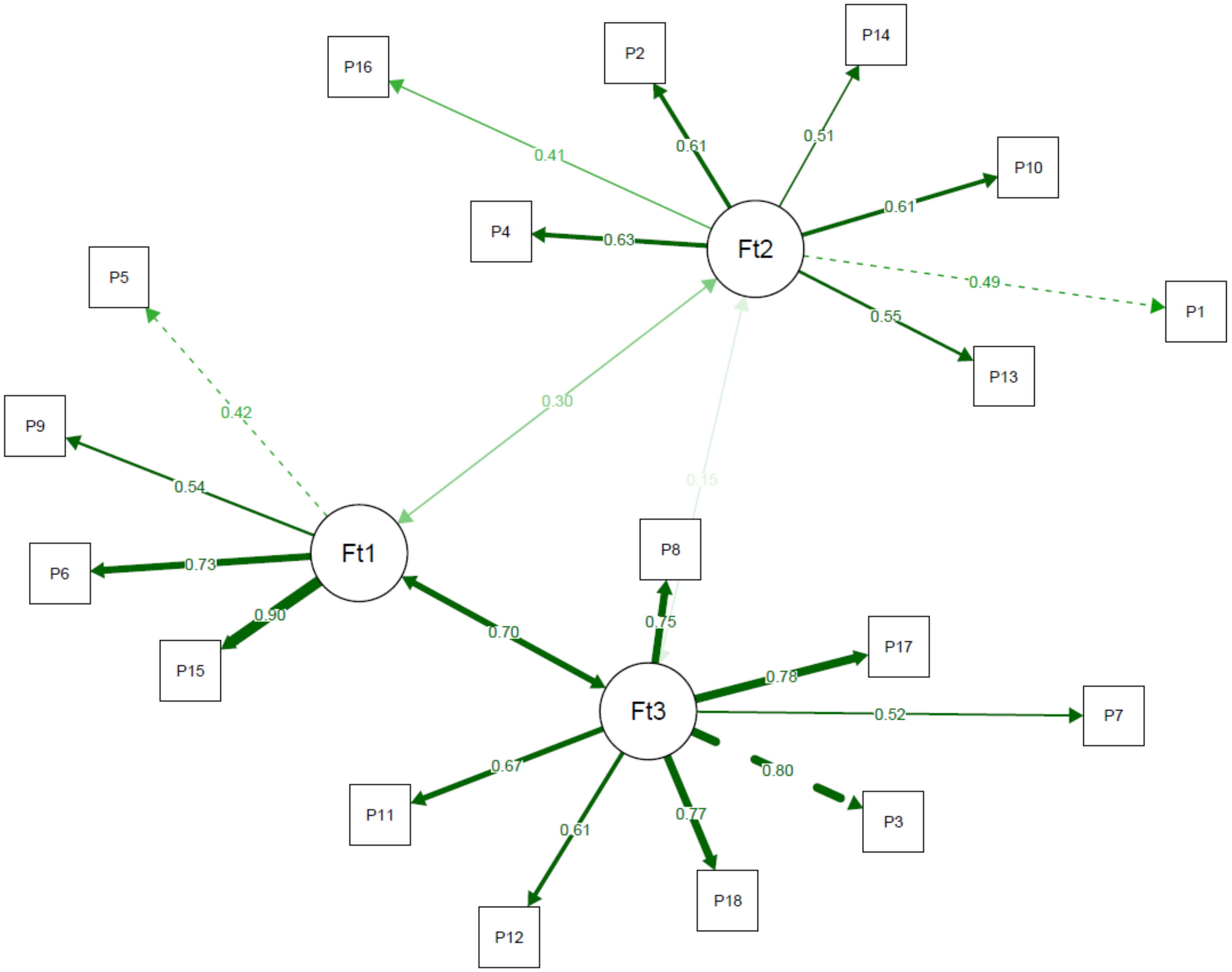

Figure S11. **Factor structure of the right political network (with edges representing factor loadings).**

Table S5. Results from Confirmatory Factor Analyses (CFAs) on each political network (using WLSM estimator).

Factor Model	SRMR	CFI	TLI	RMSEA	NFI
Left Political Network	0.08	0.93	0.92	0.07	0.91
Centre Political Network	0.09	0.82	0.79	0.08	0.81
Right Political Network	0.09	0.91	0.89	0.09	0.88

Note. WLSMV = CFA weighted least squares estimator with mean- as well as variance-corrected test statistics and robust standard errors; SRMR = Standardized Root Mean Square Residual; CFI = Comparative Fit Index; RMSEA = Root Mean Square Error of Approximation; NFI = Normed Fit Index.

Section V: Network Replicability

Following recent recommendations in the network psychometrics literature (Williams et al., 2019), our three political networks were estimated using different hyperparameter values. As outlined in Section I, the gamma value of the EBIC determines the final model sparsity, with higher gamma values being resultant in more sparse network structures (and thereby have a preference of specificity over discovery). Since our main networks were estimated using high gamma values, in this network replicability section, we explore the results of lower gamma values (which result in more dense network structures and therefore have a preference for sensitivity over specificity). It has been noted in previous literature that although more prone to Type I errors (i.e., false-positive findings), low gamma values in the EBIC glasso network estimation procedure tend to maximize differences between the network structures of different populations (e.g., van Borkulo et al., 2015; Boschloo, van Borkulo, Borsboom, & Schoevers, 2016). For this reason, we were particularly interested and intrigued to examine whether the differences we had observed between the three political networks in our main study were not only replicated but also exacerbated under more liberal statistical procedures.

Our results revealed that this is partly the case here. In particular, our supplementary network comparison tests replicated the main patterns of the paper (i.e., significant differences between edges, centrality indices, and the global strength/expected influence), with the addition of other patterns. These results are too numerous to be outlined here, but the interested reader can find them online at our OSF repository: https://osf.io/uxa32/.

Section VI: Centrality

For completeness of results, we have estimated another two ‘centrality metrics’:

betweenness and closeness.

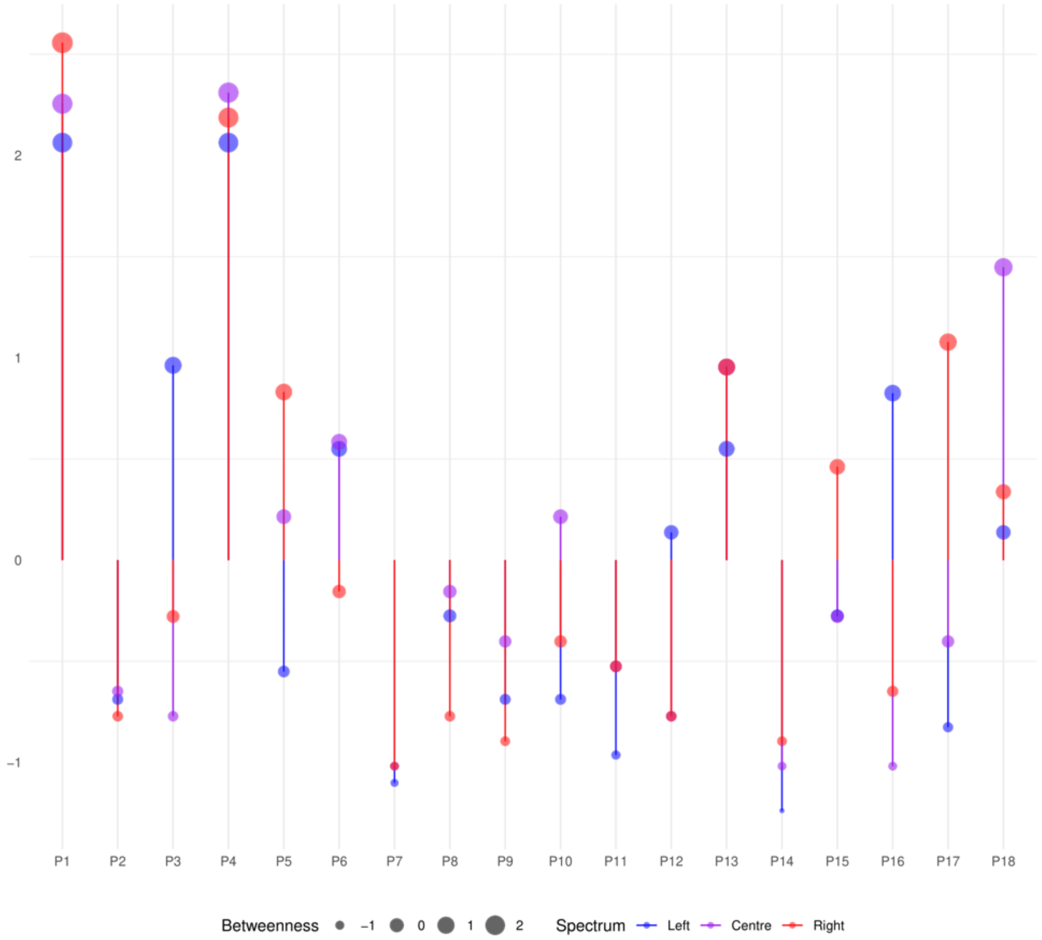

Figure S12. **Betweenness indices:** These indices reflect the frequency with which certain nodes (variables) lie in-between two other nodes (variables) in the network.

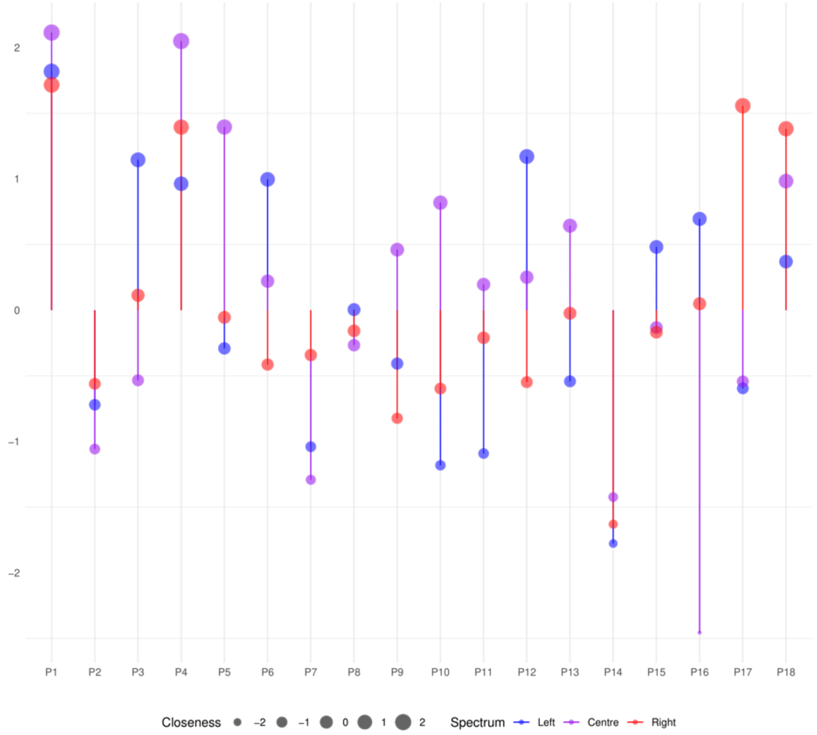

Figure S13. **Closeness indices:** These indices reflect the extent to which a node (variable) is closely related to the rest of the nodes (variables) in the network.

References

Borsboom, D. (2017). A network theory of mental disorders. *World psychiatry*, *16*(1), 5-13.

Boschloo, L., van Borkulo, C. D., Borsboom, D., & Schoevers, R. A. (2016). A prospective study on how symptoms in a network predict the onset of depression. *Psychotherapy and psychosomatics*, *85*(3), 183-184.

Brush, S. G. (1967). History of the Lenz-Ising model. *Reviews of modern physics*, *39*(4), 883.

Chen, J., & Chen, Z. (2008). Extended Bayesian information criteria for model selection with large model spaces. *Biometrika*, *95*(3), 759-771.

Christensen, A. P., & Golino, H. (2019). Estimating the stability of the number of factors via Bootstrap Exploratory Graph Analysis: A tutorial. *PsyArXiv*, *10*.

Dalege, J., Borsboom, D., van Harreveld, F., & van der Maas, H. L. (2018). The attitudinal entropy (AE) framework as a general theory of individual attitudes. *Psychological Inquiry*, *29*(4), 175-193.

Dalege, J., Borsboom, D., van Harreveld, F., & van der Maas, H. L. (2019). A network perspective on attitude strength: Testing the connectivity hypothesis. *Social Psychological and Personality Science*, *10*(6), 746-756.

Dalege, J., Borsboom, D., Van Harreveld, F., Van den Berg, H., Conner, M., & Van der Maas, H. L. (2016). Toward a formalized account of attitudes: The Causal Attitude Network (CAN) model. *Psychological review*, *123*(1), 2.

Dalege, J., Borsboom, D., van Harreveld, F., Waldorp, L. J., & van der Maas, H. L. (2017). Network structure explains the impact of attitudes on voting decisions. *Scientific reports*, *7*(1), 1-11.

Epskamp, S., Borsboom, D., & Fried, E. I. (2018b). Estimating psychological networks and their accuracy: A tutorial paper. *Behavior Research Methods*, *50*(1), 195-212.

Epskamp, S., Maris, G., Waldorp, L., Borsboom, D., Irwing, P., Hughes, D., & Booth, T. (2018a). Handbook of psychometrics. *Network psychometrics. New York, NY: Wiley-Blackwell*.

Finnemann, A., Borsboom, D., Epskamp, S., & van der Maas, H. L. (2021). The Theoretical and Statistical Ising Model: A Practical Guide in R. *Psych*, *3*(4), 594-618.

Gawronski, B. (2012). Back to the future of dissonance theory: Cognitive consistency as a core motive. *Social cognition*, *30*(6), 652-668.

Haslbeck, J. M., Epskamp, S., Marsman, M., & Waldorp, L. J. (2021). Interpreting the Ising model: The input matters. *Multivariate behavioral research*, *56*(2), 303-313.

Hirsh, J. B., Mar, R. A., & Peterson, J. B. (2012). Psychological entropy: a framework for understanding uncertainty-related anxiety. *Psychological review*, *119*(2), 304.

Jaynes, E. T. (1965). Gibbs vs Boltzmann entropies. *American Journal of Physics*, *33*(5), 391-398.

Kauffman, S. A. (1993). *The origins of order: Self-organization and selection in evolution*. Oxford University Press, USA.

Kruis, J. (2020). Transformations of mixed spin-class Ising systems. *arXiv preprint arXiv:2006.13581*.

Robinaugh, D. J., LeBlanc, N. J., Vuletich, H. A., & McNally, R. J. (2014). Network analysis of persistent complex bereavement disorder in conjugally bereaved adults. *Journal of abnormal psychology*, *123*(3), 510.

Schrödinger, E. (2007). What is life? Cambridge: Cambridge University Press.

Tibshirani, R. (1996). Regression shrinkage and selection via the lasso. *Journal of the Royal Statistical Society: Series B (Methodological)*, *58*(1), 267-288.

van Borkulo, C. D., van Bork, R., Boschloo, L., Kossakowski, J. J., Tio, P., Schoevers, R. A., ... & Waldorp, L. J. (2022). Comparing network structures on three aspects: a permutation test. *Psychological methods*.

van Borkulo, C., Boschloo, L., Borsboom, D., Penninx, B. W., Waldorp, L. J., & Schoevers, R. A. (2015). Association of symptom network structure with the course of depression. *JAMA psychiatry*, *72*(12), 1219-1226.

Williams, D. R., Rhemtulla, M., Wysocki, A. C., & Rast, P. (2019). On nonregularized estimation of psychological networks. *Multivariate behavioral research*, *54*(5), 719-750.
