# Supplementary material for: The structure of mass political belief systems: A network approach to understanding the left-right spectrum
Source: PLoS One. 2025 Oct 22;20(10):e0333595. doi: 10.1371/journal.pone.0333595 (PMC12543193; doi:10.1371/journal.pone.0333595)
Supplement: Attachment — Submitted filename: Rebuttal letter(Bentall) April25.docx [file pone.0333595.s003.docx]

**PONE-D-24-34416 Rebuttal letter**

The authors would like to thank the two reviewers for their diligence in reviewing our manuscript and especially for the detailed and very helpful suggestions by Reviewer 2, who identifies himself as Dr S. Adil Saribay. We have tried to respond to the reviewers’ recommendations as much as possible, and we feel confident that, thanks to their advice, our paper is now much stronger.

**Reviewer 1**

1. This reviewer asks us to include the Baldassari and Goldberg (2014) paper in the background material included in our introduction

RESPONSE: We are grateful for the referee for drawing our attention to this study which is indeed relevant to our own work. The approach used in the study is highly complex and somewhat different from our own, but we have provided a brief summary of the main finding on page 9 of the revised manuscript.

2. We are asked to provide an expanded explanation of network methods that will be more comprehensible to people who are not familiar with the approach.

RESPONSE: We have expanded our explanation of network approaches on pages 10 and 11 of the manuscript in a way that will hopefully make the approach easier to grasp for someone who has no familiarity with these methods.

3. This reviewer argued that an additional robustness check would be valuable: “The authors divide the public based on a 10-point self-identified ideology measure, and then estimate 3 networks. What do the results look like when what counts as a middle category (moderates) is changed to just 5,6? What happens when the left and right are broadened/narrowed? In other words, are the differences in structure there if the authors play with how they split the data initially?”

RESPONSE: Many thanks for recommending this excellent robustness check. We had in fact previously explored a similar sensitivity check, namely whether our results held if we randomly selected different subpopulations from each of the three groups (i.e., random and equal samples for left-wing, centrist – defined as scoring 4 to 7 on the measure of ideology – and right-wing populations). We had reported these findings in our Supplement Section III and showed that they pointed to the exact same results: namely, that centrist populations exhibited weaker network connectivity compared to the left-wing and right-wing.

We have now repeated the same procedure for the centrist population defined by scores of 5 to 6, rather than 4 to 7, as suggested by this referee. We found that our main result (significantly higher network connectivity in the left-wing and right-wing populations versus the centrist populations) remains invariant in this case. We have added the results of this new analysis in our Supplement Section III and comment on them in our Results section on page 23 and in the discussion on page 32.

**Reviewer 2**

1. This reviewer says that the distinction between interpersonal and intrapersonal liberalism is problematic because the terms interpersonal and intrapersonal do not closely match “society-focused” and “personal”, respectively. He makes the interesting suggestion that the common thread in what we termed the “intrapersonal” cluster is that all of the issues involve the right to make fundamental personal choices about one's body, identity, or life, and that the elements in the “interpersonal” cluster have a broader scope but consistently emphasizes international cooperation and social support rather than isolation or punishment. The referee also thinks that our use of the term “woke” is not appropriate for an academic paper.

RESPONSE: While the labelling of the communities is to some extent a subjective issue, we are happy to relabel the two contested communities as “personal liberalism” and “altruism and cooperativeness” (page 23 of the results and pages 29 in the discussion). We also agree that it was a mistake to use the term ‘woke’, and we have therefore removed it from the manuscript.

2. The reviewer says: “Perhaps the most interesting finding is that the data-driven approach yields three clusters of attitudes that do not map onto social and economic conservatism. But I wonder if this might have anything to do with the list of issues used in data collection. The manuscript does not inform us who else used this or a similar list in their research (apart from Wilson in 1968) and say anything about reliability and validity. Only four of the issues seem to be more about economic than social conservatism (P2, P7, P10, P16—the latter assuming Brexit is primarily about economic sovereignty/regulation). I think 9 of the issues are clearly about social conservatism and the remaining 5 are a mix. Would the authors agree with this and if so, is this imbalance of issue representation relevant to the divergence of their findings from the literature?”

RESPONSE: The items were based on Wilson’s measure, which the authors adapted to include items that reflect issues that seem important at the present time.

The validity of the items is established by the fact that the three groups, Left, Centre and Right, differed on them exactly in the way expected as shown in Table 1, and we have now added a comment to this effect on page 32 of the discussion. The reliability of the measure is not relevant to our analysis because network psychometrics do not assume that the items belong to a scale (i.e., that they are indicators of an underlying latent trait), but nonetheless, it has been possible to calculate a reliability coefficient with the direction of each item determined by the group differences, and it is high, 0.83. We now note this on the scale description in the methods section, page 15.

We agree with this reviewer’s suggestion that the communities that emerged may have been affected by the balance between economic and social items. We now acknowledge this in the discussion on page 31.

3. The reviewer requests more detail about the childrearing scale and asks us to check our naming of it throughout.

RESPONSE: This scale is a very widely used measure in political science. We have expanded the description of its origins in the methods section, page 15, and also calculated its reliability which is marginal, although we do not think this is a critical weakness as the scale is only used in a supporting analysis as a way of validating our community structure. We acknowledge this low reliability in the limitations section on page 32.

4. The referee asks us to explain how our findings relate to the large literature on attitudes strength, perhaps in a footnote.

RESPONSE: The literature on attitude strength is, as the referee states, very large and therefore beyond the scope of our paper. However, in our submitted manuscript we discussed this implicitly by commenting on how our findings are consistent with research on the certainty and resistance to change of political attitudes. We have slightly expanded this paragraph on page 30 of the revised discussion section to make this point clearer and to link to Dalege’s work on causal attitude networks.

5. The referee asks us to make it clearer how and why Converse’s approach (“beliefs as a network”) “contrast sharply” with factor-analytic approaches.

RESPONSE: We think our rewritten account of network approaches, made in response to Referee #1’s point 2 goes some way to addressing this issue. However, we have removed the word ‘sharply’ from the first paragraph of our account of Converse.

6. The referee asks us to provide more information in the methods about how representative the sample is with regard to important aspects of the UK adult population more concretely.

RESPONSE: We report that our sample was stratified by age, sex and household income in quotas that represented the structure of the UK population according to the UK Office of National Statistics. We also report the proportion who were female, the proportion who came from each of the constituent administrations in the UK, the proportion who were graduates, the proportion who describe themselves as working class, and the sample’s voting behaviour in the Brexit referendum. In two instances (the proportion of graduates and voting) we report that our sample has drifted from the comparable population figures because of attrition across survey waves. We have added a reference to the latest Office of National Statistics population report (page 14) but, otherwise, we do not understand how we can make our account of our sample more concrete than it is.

7. The referee points out that he abstract reports sample size as 1,634 but on p. 13, we learn that it is 1,643 + 415.

RESPONSE; We thank the referee for pointing this out. The abstract was in error and has been corrected.

8. The reviewer asks us to report patterns of missing data.

RESPONSE: There was no missing data because our survey software required responses for every item before moving on. This is now mentioned at the beginning of the measures section.

9. This reviewer suggests that we include effect sizes in Table 1

RESPONSE: After some thought, we have decided not to include effect sizes because the figure is crowded as it is and because these are not relevant to the hypotheses that we have tested in our paper.

10. This reviewer has pointed to many issues with our figures, partly due to my accidental inclusion of the wrong versions of the figures in my last submission. He makes many important and useful observations and we are very grateful for his diligence and the opportunity to improve them:

“Nearly every aspect of the network visualizations is unfamiliar to the majority of readers in Psychology (I’m not sure about other fields but probably the same). So, a comprehensive note underneath these figures seems like a good idea to me.”

RESPONSE: We completely agree that there should be a comprehensive figure caption to explain what each figure illustrates. We have now updated all of our figures in light of the comments and included a detailed caption to more clearly explain them.

“At the least, in Figure 1, the vertical axis on the top panel could be labelled either on the figure or in a note. I assume that this axis is edge-weights. I’m also not sure what the lines in the figure (as opposed to circles) represent; maybe confidence intervals of estimated edge-weights, though I’m not sure why those would not be symmetrical. I assume the centre and left circles overlap completely in P1. Perhaps they could be jittered. For the bottom panel, I think the “pie around each node” represents node predictability. This is explained in the text but is disconnected from the figure. It’s better to place this in a note below the figure.”

RESPONSE: Again, thanks for these suggestions! We have now completely redrawn our figures to make it clearer to the readers that we are presenting three key findings: first and foremost, the three network structures for the left, centre, right; second, the centrality of each node within the network; finally, the network comparison tests. (We apologise that this was often unclear our previous submission, e.g., that the line graph shown at the top of our previous Figure 1, was meant to represent the centrality of each node; we no longer include this in the present submission). We also include a comprehensive caption underneath that states what each panel illustrates. We hope this major update addresses the reviewer’s point.

“In Figure 2, it seems that the top left network represents leftists, the top right one represents centrists, and the bottom network represents rightists. Labelling these networks would be better. What is the difference from the bottom panel of Figure 1 apart from the additional representation of community structure. These two sets of 3 networks seem redundant. If this is correct, I would remove the bottom panel of Figure 1.”

RESPONSE: We agree that it is redundant to display the same networks twice. We have thus excluded the networks from Figure 2 and simply outline the factor structure of our three networks.

“The panels in Figure 1 are called Figure 1a (I assume the top panel) and 1b (the bottom panel) in the text but not labelled in the figure. A large “A” or “B” next to these panels and/or a note should be added.”

RESPONSE: Many thanks for catching this omission from our end! We have now included large (A), (B), and (C) letters to clearly outline the three panels of Figure 1.

“The authors mention that Figure 1b shows “that a particular node scores the highest for the Left and Centre networks, namely, P6 (“Gay Rights”). By contrast, node P17 (“Public Demonstrations”) scored the highest in the Right network.” I assumed that figure 1b was the bottom panel but looking at this figure, what I see is that line thickness varies significantly between connections and represents connection strength; some thin connections might have very low weights while thicker ones have much higher weights; and P6 appears to have several relatively thick connections especially in Leftists. However, it's not immediately apparent that P6's connection weights would sum to a higher total than nodes like P3 or P12, which appear to have both numerous and thick connections. Without the actual edge weight data, we can't verify their claim about P6 having the highest strength centrality. If Figure 1b is the top panel, then once again, I cannot see how P17 is the most central node for rightists. There are rightist nodes higher on the Y-axis and circle size does not help here. I did not see any data in the Supplementary Information that could help with this. Perhaps I’m missing something due to not being so familiar with network analyses but whatever I’m missing could also be missed by other readers. So, further explanation would be helpful.”

RESPONSE: Thank you for noting that this part of the text and figure was unclear. To address this, we have first and foremost revised entirely Figure 1 to more clearly display these results: our updated line graph clearly shows that P6 scores the highest (but we agree with the reviewer that other nodes, like P3 and P12, score high as well, and this is now better visualized). Second, we have included a caption underneath Figure 1 to explain what each panel illustrates (for example, the P17 node now can more clearly be seen to be highest for the right-wing population). Finally, we have also updated parts of our results section to more clearly explain these patterns. We hope these major changes clarify that what these centrality scores indicate.

Finally, the reviewer says, “In Table 2, we are presented with “predictability indices ... that are highest uniquely for each network.” I understand the general approach but do not know if there is a convention in network analysis for deciding on these or whether the authors had their own specific criteria. This could be clarified.”

RESPONSE: There are no guidelines for choosing the highest predictability index. However, this result is purely descriptive and not meant to *inferentially* adjudicate clear differences across the three populations (as centrality indices, for instance, were meant to do). We now clarify these points in our interpretation and outline of results.

As I hope will be obvious, we have gone to some considerable efforts to revise our manuscript in the light of the comments of both reviewers, and we believe that the resulting paper is both stronger and clearer as a consequence. We hope that it is now suitable for publication in PLoS One.

Sincerely

Richard Bentall
